# Supplementary material for: Identification and functional analysis of non-coding regulatory small RNA FenSr3 in Bacillus amyloliquefaciens LPB-18
Source: PeerJ. 2023 May 15;11:e15236. doi: 10.7717/peerj.15236 (PMC10194069; doi:10.7717/peerj.15236)
Supplement: Supplemental Information 4 [file peerj-11-15236-s004.zip › KO/CK-vs-T1_map/map04022.html]

KEGG PATHWAY: cGMP-PKG signaling pathway - Reference pathway


|  |  |
| --- | --- |
| **cGMP-PKG signaling pathway - Reference pathway** |  |

[
Pathway menu
| Organism menu
| Pathway entry
| Show description
| User data mapping
]

Cyclic GMP (cGMP) is the intracellular second messenger that mediates the action of nitric oxide (NO) and natriuretic peptides (NPs), regulating a broad array of physiologic processes. The elevated intracellular cGMP level exerts its physiological action through two forms of cGMP-dependent protein kinase (PKG), cGMP-regulated phosphodiesterases (PDE2, PDE3) and cGMP-gated cation channels, among which PKGs might be the primary mediator. PKG1 isoform-specific activation of established substrates leads to reduction of cytosolic calcium concentration and/or decrease in the sensitivity of myofilaments to Ca2+ (Ca2+-desensitization), resulting in smooth muscle relaxation. In cardiac myocyte, PKG directly phosphorylates a member of the transient potential receptor canonical channel family, TRPC6, suppressing this nonselective ion channel's Ca2+ conductance, G-alpha-q agonist-induced NFAT activation, and myocyte hypertrophic responses. PKG also opens mitochondrial ATP-sensitive K+ (mitoKATP) channels and subsequent release of ROS triggers cardioprotection.

|  |  |  |
| --- | --- | --- |
| Reference pathway | 184% 150% 122% 100% 82% 67% 55% | 图片下载 |
